# NIH workshop report on the trans-agency blood–brain interface workshop 2016: exploring key challenges and opportunities associated with the blood, brain and their interface

**DOI:** 10.1186/s12987-017-0061-6

**Published:** 2017-05-01

**Authors:** Margaret J. Ochocinska, Berislav V. Zlokovic, Peter C. Searson, A. Tamara Crowder, Richard P. Kraig, Julia Y. Ljubimova, Todd G. Mainprize, William A. Banks, Ronald Q. Warren, Andrei Kindzelski, William Timmer, Christina H. Liu

**Affiliations:** 1 National Heart, Lung, and Blood Institute, National Institutes of Health, 6701 Rockledge Dr., Room 9149, Bethesda, MD 20892-7950 USA; 2 Zilkha Neurogenetic Institute, Los Angeles, CA USA; 3 Johns Hopkins School of Medicine, Baltimore, MD USA; 4 Combat Casualty Care Research Program, Fort Detrick, MD USA; 5 University of Chicago Medical Center, Chicago, IL USA; 6 Cedars-Sinai Medical Center, Los Angeles, CA USA; 7 Sunnybrook Health Science Centre, Toronto, ON Canada; 8 University of Washington, Seattle, WA USA; 9 National Cancer Institute, National Institutes of Health, Bethesda, MD USA

**Keywords:** Blood–brain barrier, Extracellular vesicles, Exosomes, Cancer, Neurodegeneration, Traumatic brain injury, Therapeutics, Delivery

## Abstract

A trans-agency workshop on the blood–brain interface (BBI), sponsored by the National Heart, Lung and Blood Institute, the National Cancer Institute and the Combat Casualty Care Research Program at the Department of Defense, was conducted in Bethesda MD on June 7–8, 2016. The workshop was structured into four sessions: (1) blood sciences; (2) exosome therapeutics; (3) next generation in vitro blood–brain barrier (BBB) models; and (4) BBB delivery and targeting. The first day of the workshop focused on the physiology of the blood and neuro-vascular unit, blood or biofluid-based molecular markers, extracellular vesicles associated with brain injury, and how these entities can be employed to better evaluate injury states and/or deliver therapeutics. The second day of the workshop focused on technical advances in in vitro models, BBB manipulations and nanoparticle-based drug carrier designs, with the goal of improving drug delivery to the central nervous system. The presentations and discussions underscored the role of the BBI in brain injury, as well as the role of the BBB as both a limiting factor and a potential conduit for drug delivery to the brain. At the conclusion of the meeting, the participants discussed challenges and opportunities confronting BBI translational researchers. In particular, the participants recommended using BBI translational research to stimulate advances in diagnostics, as well as targeted delivery approaches for detection and therapy of both brain injury and disease.

**Electronic supplementary material:**

The online version of this article (doi:10.1186/s12987-017-0061-6) contains supplementary material, which is available to authorized users.

## Background

The goal of the trans-agency blood–brain interface (BBI) workshop was to bring together experts to examine key challenges and develop recommendations for further studies of the interface between the circulatory system and the brain. The interagency workshop was designed to include sessions on the interface itself as well as sessions on blood biomarkers, targeted drug delivery, and brain disorders that release biomarkers into the circulation (Additional file 1).

A common scientific theme that has recently emerged is the role of the immune system and the inflammasome (e.g., innate immune system sensors for immediate response to molecular signals from the injured site or invading microbes). Another emerging area is the role of extracellular vesicles (EV), including exosomes and microparticles, and how they can be harnessed for both diagnostic and targeted drug delivery applications. It should be noted that the nomenclature and classification of these cell-derived vesicles is still in progress [1].

Experts in the blood sciences, BBB biology, BBB modeling and technology development—including exosome therapeutics, platform technologies, and nanotechnology—were invited to the workshop to present their latest ideas and research. The workshop presentations and ensuing interdisciplinary discussions highlighted the potential of technologies for diagnostics and therapy development and the need for collaborations across the various groups represented at the meeting. The workshop concluded by generating a set of recommendations to facilitate future development in this promising yet under-represented field.

### Keynote presentation I: MRI-guided focused ultrasound surgery to open the blood–brain barrier in the treatment of brain tumors

Dr. Todd Mainprize discussed the potential of MRI-guided focused ultrasound surgery to create openings in the BBB. Dr. Mainprize noted that glioblastoma is the most malignant brain tumor, characterized by a very poor prognosis. Many therapeutic approaches have been developed to treat glioblastoma but do not improve patient survival, in part due to the lack of effective drug delivery through the BBB. Several methods have been employed to circumvent the BBB and more effectively deliver therapeutics to the brain, including direct intra-tumoral injection, osmotic disruption following intra-arterial carotid delivery, and convection-enhanced delivery.

Ultrasound has been used in medical applications since the early 1900s and has been used for therapeutic purposes in the CNS since the 1950s. Dr. Mainprize pointed out that more recently, magnetic resonance-guided focused ultrasound (MRgFUS), which is a non-invasive procedure that reversibly opens the BBB without damage to the surrounding neurons, has been employed for targeted drug delivery to the brain [2]. Recent studies involving non-human primates [3] investigated the effectiveness and characteristics of BBB opening by MRgFUS. A clinical trial, designed to establish the feasibility, safety and preliminary efficacy of MRgFUS to open the BBB for the delivery of chemotherapeutic agents in brain tumors, has been approved by Health Canada. This single-site Phase I study (NCT02343991), is currently recruiting patients (n = 6 max) at the University of Toronto, ON, Canada. Preliminary results have shown an increase in gadolinium uptake (indicative of BBB opening) in the sonicated area of the brain. Treatment with intravenous (IV) doxorubicin, which has very poor oral bioavailability, showed a two to threefold increase in concentration via targeted delivery to the brain tumor. Dr. Mainprize cautioned that the results of this limited interventional trial are preliminary, but suggest that MRgFUS may provide a new method to deliver therapeutic agents into the brain.

### Blood science session: Part I (Fig. 1)

#### The blood–brain barrier and neurodegeneration

**Fig. 1:**
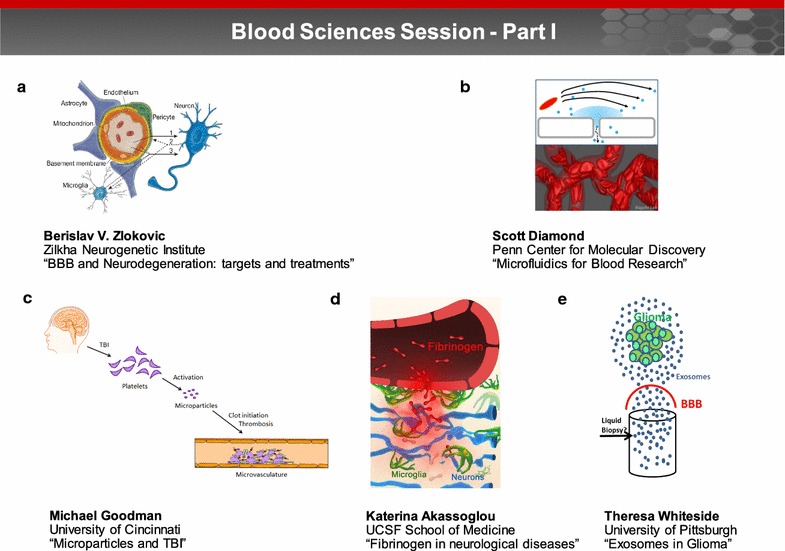
Blood sciences session—Part I. Part I of the blood sciences session focused on the physiology of the blood and neurovascular unit, biofluid-based molecular markers, and extracellular vesicles associated with brain injury and disease progression. The presentations explored how these entities are transported between brain and blood and can be employed to evaluate injury states or deliver therapeutics. **a** Endothelial cells, pericytes, neurons and glia constitute an interconnected functional cellular network, collectively referred to as the neurovascular unit. Dr. Berislav Zlokovic discussed how defects in blood vessels and the neurovascular unit can lead to BBB breakdown and neurodegeneration (image reprinted with permission from [107]). **b** The Diamond laboratory uses a combination of microfluidics, patient-specific high throughput phenotyping, and systems biology to quantify blood function in the hemodynamic and pharmacological context of thrombosis and hemostasis. Image courtesy of Dr. Diamond. **c** Dr. Michael Goodman discussed how traumatic brain injury (TBI) induces systemic alterations in the aggregation of platelets aggregation and as well as the generation and function of microparticles (MP). Image courtesy of Dr. Goodman. **d** Dr. Katerina Akassoglou identified fibrinogen as a novel molecular link between blood–brain barrier disruption, activation of CNS innate immunity, and neurodegeneration. Image courtesy of Dr. Akassoglou. **e** Dr. Theresa Whiteside noted that genetic and molecular cargo of exosomes found in plasma of glioma patients resembles that of tumor cells and suggested that tumor derived exosomes hold promise as biomarkers of prognosis, as a source of liquid biopsy. Image courtesy of Dr. Whiteside

Blood vessels in the brain are organized in parallel with the major neurologic circuits tasked with sensation, memory and motion. Tight interrelationships between these circuits may reflect key functional roles the vasculature plays in normal neuronal function, disease and aging [4]. Dr. Berislav Zlokovic discussed the role of the BBB in neurologic disorders, with an emphasis on targets and treatments. He discussed: (1) how defects in blood vessels and the neurovascular unit can lead to BBB breakdown and neurodegeneration (Fig. 1a) [4], (2) the effects of genetic risk factors for Alzheimer’s disease (AD) on blood vessels and BBB [5], and (3) the importance of new neurovascular imaging techniques and biomarkers in predicting cognitive impairment [6]. Potential therapeutic targets and treatments directed at the neurovascular unit in AD and stroke were described, including inhibition of the receptor for advanced glycation end-products in AD [7] and protection of the BBB and the neurovascular unit by protease activated protein C in stroke.

#### Microfluidics for blood research: from disease simulation to patient-specific phenotyping to diagnostics

Dr. Scott Diamond discussed how the quantitation of blood function during thrombosis and hemostasis can be achieved through a combination of systems biology, patient-specific high throughput phenotyping, as well as microfluidics, which is a multidisciplinary field that focuses on the science and technology of fluid flow on a submillimeter scale (Fig. 1b) [8, 9]. Advances in anticoagulation techniques allow the study of platelet function alone, platelet and thrombin function, and thrombosis via both the contact and extrinsic pathways [10]. In addition to blood function, Dr. Diamond’s laboratory also includes studies on the interaction of patient blood with cultured endothelium in microfluidic environments. This is part of a larger effort to recreate the neurovascular unit while maintaining flow and measuring the resistivity of the system. However, the phenotypic variability and lack of standardization of cultured human brain endothelium remains a challenge. Dr. Diamond explained that the development of endothelial permeability assays in vitro will help facilitate the evaluation of trans-endothelial transport mechanisms under flow conditions. Compared to animal models, microfluidics offers a highly reproducible and translatable model with improved control and visualization capabilities. Dr. Diamond concluded that microfluidic systems can also aid in optimization of nanoparticle formulations, measuring changes in blood chemistry and platelet function during trauma [11] or stroke, and the determination of novel cellular signaling pathways that regulate the BBB.

#### Microparticles impact coagulation after traumatic brain injury

Dr. Michael Goodman discussed how traumatic brain injury (TBI) induces systemic alterations in the aggregation of platelets as well as the generation and function of microparticles (MP), which are cell-derived extracellular vesicles (EVs) in the 0.1–1 µm range (Fig. 1c) [12]. Dr. Goodman began by noting that the pathophysiology that drives the subacute, persistent hypercoagulable state commonly seen after TBI is not well understood. Alterations in platelet and MP numbers and function caused by TBI, have been suggested as possible causes; however, the contributions of platelets and MPs are currently unknown. Dr. Goodman emphasized that further opportunities remain to determine the direct effect of injury-derived MPs on platelet function and endothelial activation. Studies examining the ability of MPs to traverse the BBB have yet to be performed. Future research will need to focus on the multitude of physiologic and pathologic functions of specific cell-derived MPs in response to injury [13–16].

#### Fibrinogen in neurological diseases: mechanisms, imaging, therapeutics

A key challenge for the BBB field is the limited understanding of the consequences of blood proteins in the CNS and their contribution to neuronal dysfunction. Protection of the CNS from leakage of plasma proteins by the BBB is compromised in a wide range of neuroimmune and neurodegenerative diseases, as well as after TBI. Dr. Katerina Akassoglou identified fibrinogen as a novel molecular link between blood and brain barrier disruption (BBBD), activation of CNS innate immunity, and neurodegeneration (Fig. 1d) [17]. Following fibrinogen conversion to pro-inflammatory fibrin, the CD11b/CD18 integrin receptor (also known as Mac-1 and complement receptor 3) is activated on microglial cells [18, 19]. Genetic disruption of the fibrin/CD11b interaction suppresses this microglial activation process and results in a reduction of neurologic symptoms, inflammation, demyelination, and axonal damage in experimental autoimmune encephalomyelitis (EAE) [17, 20, 21]. Dr. Akassoglou concluded that targeting the functions of fibrin in the CNS without affecting its beneficial effects in hemostasis holds promise for therapeutic intervention, and suggested that studies of fibrin-induced neuroinflammation in neurological diseases associated with fibrin deposition and microglial activation, such as TBI and AD, are also warranted.

#### Exosomes in glioma: their potential as carriers of information between the tumor and immune cells

Exosomes, the smallest (30–150 nm) of cell-derived extracellular vesicles (EVs) are present in all body fluids and serve as carriers of information between tumors, immune cells and normal tissue cells. Dr. Theresa Whiteside presented her research involving exosomes in glioma. Dr. Whiteside noted that genetic and molecular cargo of exosomes found in plasma of glioma patients resembles that of tumor cells and suggested that tumor derived exosomes hold promise as biomarkers of prognosis, as a source of liquid biopsy (Fig. 1e). Studies determined that changes in total exosomal protein and mRNA levels could serve as surrogate markers of immunological and clinical responses in glioma patients receiving anti-tumor vaccines [22]. Results from a Phase I/II clinical trial (NCT00766753) indicated that protein and mRNA expression levels for immune-related genes in exosomes (isolated from glioma patients’ plasma) were useful in predicting glioma patients’ response to dendritic cell (DC)-based vaccination therapy, and could potentially serve as surrogate markers of anti-tumor immune activity and survival.

### Blood science session: Part II (Fig. 2)

BBB integrity is critical for preserving brain function following injury (e.g., TBI). Dr. Tamara Crowder emphasized the unique set of challenges for managing BBB integrity in combat-related TBI. Treatment of combat casualties is often complicated by polytrauma and medics must balance lifesaving measures for TBI in combination with hemorrhage, burn, and/or amputation to give the casualty the best chance of survival. The primary goal of the Defense Health Agency’s Neurotrauma program is to decrease TBI-related morbidity and mortality, mitigate secondary brain injury, and to facilitate interventions and capabilities for improved patient care. Tools and technologies are currently being developed to improve capabilities for assessment and monitoring of CNS trauma to: (1) explore the relationship among the post-traumatic cerebral blood, autoregulation and the neurovascular unit; and (2) leverage different transporters at the BBIs to regulate the brain metabolomics and pharmacologic microenvironment. According to Dr. Crowder, these tools and technologies offer opportunities to improve clinical practice following brain injury.

**Fig. 2:**
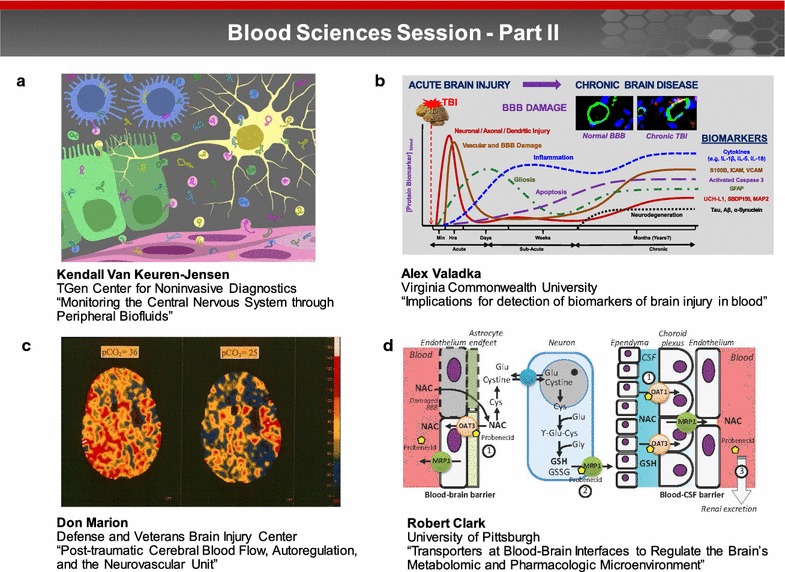
Blood sciences session—Part II. Tools and technologies are currently being developed to improve capabilities for assessment and monitoring of CNS trauma to: (1) explore the relationship among the post-traumatic cerebral blood, autoregulation and the neurovascular unit; and (2) leverage different transporters at the BBIs to regulate the brain metabolomics and pharmacologic microenvironment. **a** Dr. Kendall Van Keuren-Jensen discussed the need for accessible biomarkers to allow more frequent monitoring of the CNS and the potential to improve patient care. The presence of RNA in body fluids and its relative stability when transported via EVs or carrier proteins has captured significant attention as a source for biomarker discovery. Image courtesy of the NIH exRNA Communication Program. **b** Dr. Alex Valadka noted that differences in markers released by neuronal and glial cells may make possible the diagnosis of specific subtypes of TBI based on distinct patterns and ratios of glial and neuronal markers. Image courtesy of Dr. Valadka. **c** Brain trauma can result in an immediate decrease in the cerebral blood flow, resulting in loss, or fluctuations in multiple physiological control systems including blood pressure and chemical autoregulation. Image courtesy of Dr. Marion. **d** Dr. Robert Clark reported on the importance of membrane transporters at the BBI. These membrane transporters are responsible for efflux of exogenous substrates (e.g. drugs) at the BBB and CSF-blood barriers, impacting the brain’s microenvironment. Image courtesy of Dr. Clark

#### Monitoring the central nervous system through peripheral biofluids

The ability to monitor the CNS would advance our knowledge of disease pathogenicity, facilitate clinical care, and support the development of new therapies. Dr. Kendall Van Keuren-Jensen discussed the need for accessible biomarkers in order to allow more frequent monitoring of the CNS and the potential to improve patient care. The presence of RNA in body fluids and its relative stability when transported via EVs or carrier proteins has captured significant attention as a source for biomarker discovery (Fig. 2a). Dr. Van Keuren-Jensen’s laboratory identified CNS-enriched RNA transcripts in saliva, plasma/serum, urine, and cerebrospinal fluid (CSF). The potential of extracellular RNAs (exRNAs) for monitoring CNS diseases was demonstrated by the finding that microRNA-34c is upregulated in the hippocampus of AD patients and in mouse models of cognitive impairment [23, 24]. MicroRNA-34c was also found to be upregulated in serum and plasma from patients with dementia in two independent studies [25, 26]. The promise of exRNAs as biomarkers for the CNS is considerable; however, the field is still at an early stage in understanding their specificity and reproducibility.

#### Studying the blood–brain barrier: perspectives from understanding the biokinetics of biomarkers of brain injury

Numerous studies have documented the potential clinical utility of blood-based biomarkers of TBI. Dr. Alex Valadka noted that differences in markers released by neuronal and glial cells may make possible the diagnosis of specific subtypes of TBI based on distinct patterns and ratios of glial and neuronal markers (Fig. 2b). Most of the published literature fails to distinguish between the movement of substrates across the BBI. One resulting issue is that the presence of biomarkers that are known to appear in the blood after non-CNS trauma, or even after exercise, may be erroneously interpreted as evidence of TBI. Properly designed preclinical and clinical studies of posttraumatic changes in the biokinetics of blood-based biomarkers can significantly advance our understanding of pathological responses to TBI, both acutely and long-term [27–31].

#### Post-traumatic cerebral blood flow, autoregulation, and the neurovascular unit

Brain trauma can result in an immediate decrease in the cerebral blood flow (CBF), resulting in loss, or fluctuations in multiple physiological control systems including blood pressure and chemical autoregulation (Fig. 2c). Dr. Donald Marion noted that a significant number of severe blast TBI victims develop pseudoaneurysms and vasospasm [32–34] as the result of vascular pathology [35]. With regard to BBB integrity, reduction in CBF during hypoxia in mice closely relates to areas with compromised BBB [36]. Dr. Marion showed that there is a mismatch between cerebral metabolism and blood flow after TBI, and that such a mismatch can induce local ischemia and an increase in the penumbra surrounding the primary contusion after prophylactic hyperventilation (pCO_2_ < 35 mmHg) therapy. In addition, within contusions and the surrounding parenchyma, CO_2_ vasoreactivity may be nearly three times the normal level, suggesting hypersensitivity to hyperventilation therapy. These findings changed the acute care practice for severe TBI from aggressive hyperventilation in the 1980’s to maintaining pCO_2_ at 35-40 mmHg in 2016.

#### Employing transporters at blood–brain interfaces to regulate the brain’s metabolomic and pharmacologic microenvironment

Dr. Robert Clark reported on the importance of membrane transporters at the BBI. These membrane transporters are responsible for efflux of exogenous substrates (e.g. drugs) at the BBB and CSF-blood barriers, impacting the brain’s microenvironment (Fig. 2d). Dr. Clark stressed that many xenobiotics that cross the BBB are transported out of the brain in a dynamic and surprisingly rapid fashion. Both the impact of brain injury on transporter expression and function [37], and the impact of transporter expression and function on recovery after brain injury were discussed [38]. Importantly, there is potential to capitalize on membrane transporters to optimize brain exposure to potentially neuroprotective compounds [39, 40]. Dr. Clark’s group recently showed that co-administration of probenecid (an FDA approved antibiotic adjuvant) increases serum and brain *N*-acetyl cysteine (NAC) exposure [41]. NAC, which is also FDA approved, may be an effective countermeasure for blast-induced TBI [42]. Dr. Clark noted that repurposing combinations of transporter inhibitors/inducers and neuroprotective substrates is a promising strategy for clinical translation.

### Exosome therapeutics session (Fig. 3)

#### In vivo tracking of dendritic cell exosomes delivered to brain

**Fig. 3:**
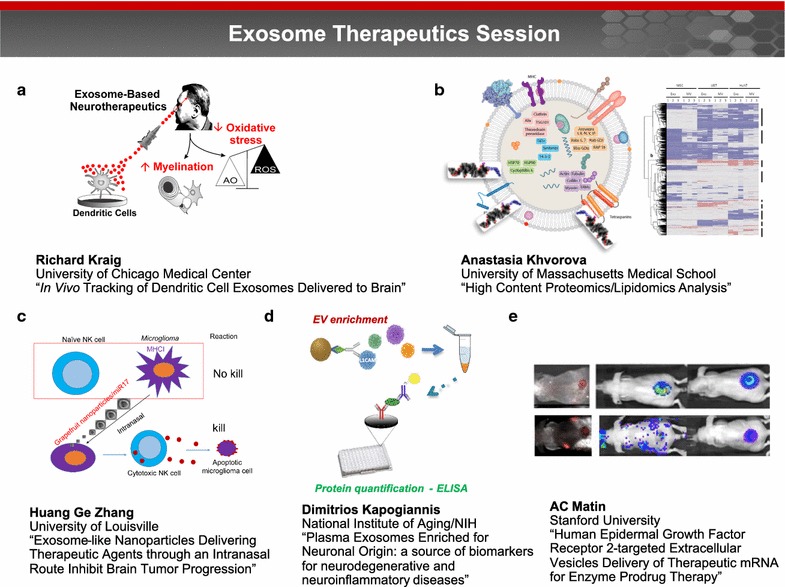
Exosome therapeutics session. Exosomes, the smallest (30–150 nm) type of cell-derived extracellular vesicles, are present in all body fluids and serve as carriers of information across the body in both health and disease. This session focused on the emerging role of exosomes, in particular how they can be harnessed for both diagnostic and targeted drug delivery applications. **a** Dr. Richard Kraig reported that interferon gamma (IFN)-stimulated dendritic cells (SDCs) release SDC-derived exosomes (SDC-exos) containing specific miRNAs which promote myelination and reduce oxidative stress when administered to brain slice cultures. Image courtesy of Dr. Kraig. **b** Dr. Anastasia Khvorova and coworkers are examining the surface lipid and protein composition of EV membranes (left image modified from [108]) through high content proteomics and lipidomics analysis to better understand how these elements contribute to EV function. Right image courtesy of Dr. Khvorova. **c** Dr. Huang Ge Zhang described a novel approach for grapefruit derived nanovector (GNV)-mediated intranasal delivery of RNA in general and therapeutic miR17 specifically to brain tumor cells as a proof of concept. miR17-mediated downregulation of MHC1 expressed on tumor cells leads to activation of Natural Killer (NK) cells and targeting of tumor cells. Image courtesy of Dr. Zhang. **d** Dr. Dimitrios Kapogiannis and coworkers developed a method for enriching peripheral blood EVs of neuronal origin using L1 cell adhesion molecule (L1CAM) immunoprecipitation. In case-controlled studies, L1CAM + EVs showed diagnostic differences for AD, and perhaps fronto-temporal dementia, multiple sclerosis, and TBI. Image courtesy of Dr. Kapogiannis. **e** By using EVs as the “body’s antigen delivery system” for targeting a novel prodrug 6-chloro-9-nitro-5-oxo-5H-benzo(a)phenoxazine (CNOB)/ChrR6 regimen specifically to HER2 positive cancer, the cytotoxic product of this regimen, 9-Amino-6-chloro-5H-benzo(a)phenoxazine-5-one (MCHB), can be visualized noninvasively in living mice. Image reprinted with permission from [58]

Dr. Richard Kraig reported that interferon gamma (IFN)-stimulated dendritic cells (SDCs) release SDC-derived exosomes (SDC-exos) containing specific miRNAs which promote myelination and reduce oxidative stress when administered to brain slice cultures (Fig. 3a) [43]. These effects emulate those seen after slice exposure to exosomes derived from the serum of animals that experienced environmental enrichment (i.e., increased physical, intellectual and social activity) [44]. According to Dr. Kraig, SDC-exos not only increase myelin levels days after nasal administration to naïve animals but also increase remyelination in vivo after lysolecithin-induced exposure, a chemical model of multiple sclerosis and reduced oxidative stress. Using fluorescently labelled SDC-exos, Dr. Kraig and his coworkers tracked SDC-exos movement into brain after nasal delivery. SDC-exos quickly entered via the olfactory route, passed along the interstitial space to the CSF and entered brain from the pial surface and perivascular space, a path consistent with that seen for soluble agents [45]. These results support the use of SDC-exos as a novel cell-based therapeutic to mitigate the impact of neurodegenerative disorders [46]. Future challenges will be to manufacture SDC-exos via standards and quantities needed for human studies.

#### High content proteomics/lipidomics analysis: on a path toward understanding the mechanisms of exosome-mediated cellular uptake and blood–brain barrier crossing

Dr. Anastasia Khvorova noted that exosomes or EVs have received much attention as vehicles for oligonucleotide (ONT) delivery, as well as endogenous carriers of disease biomarkers for diagnostic purposes. Currently, efficient non-toxic ONT delivery to the CNS represents a significant barrier to the treatment of neurological disorders, such as Huntington’s disease [47]. EVs have the potential to act as “native” ONT delivery vehicles, but robust and scalable methods for loading therapeutic RNA cargo into EVs such as exosomes are lacking. Dr. Khvorova and colleagues showed that hydrophobically modified small interfering RNA (siRNA) can be efficiently loaded into exosomes without altering vesicle size, distribution or integrity, and promote efficient neuronal internalization and *Huntingtin* RNA silencing in vitro and in vivo. Dr. Khvorova and coworkers are now examining the surface lipid and protein composition of EV membranes to better understand how these elements contribute to EV function (Fig. 3b).

#### Exosome-like nanoparticles delivering therapeutic agents through an intranasal route inhibit brain tumor progression

Dr. Huang Ge Zhang noted that although the efficacy of using mammalian cell derived exosomes and nanovectors as vehicles for intranasal delivery of therapeutic agents has been demonstrated [48, 49], biosafety, cytotoxicity considerations and large scale production have presented obstacles to their clinical use [50–52]. Dr. Zhang described a novel approach for grapefruit derived nanovector (GNV)-mediated intranasal delivery of RNA in general and therapeutic miR17 specifically to brain tumor cells as a proof of concept (Fig. 3c). The data suggest that RNA, including miR17, is effectively delivered to the brain by GNVs without observable side effects. GNVs coated with folic acid (FA-GNVs) were enhanced for targeting the GNVs to a folate receptor positive GL26 brain tumor. Intranasal administration of miR17 carried by FA-GNVs led to rapid delivery of miR17 to the brain and selective uptake by GL-26 tumor cells. Mice treated intranasally with FA-pGNV/miR17 had delayed brain tumor growth. These results demonstrate that nanovector-mediated intranasal delivery may provide a noninvasive therapeutic approach for treating brain related disease.

#### Plasma exosomes enriched for neuronal origin: a source of biomarkers for neurodegenerative and neuroinflammatory diseases

EV movement into and out of brain has considerable diagnostic and therapeutic potential for nervous system disorders. Dr. Dimitrios Kapogiannis and coworkers developed a method for enriching peripheral blood EVs of neuronal origin using L1 cell adhesion molecule (L1CAM) immunoprecipitation (Fig. 3d). In case-controlled studies, L1CAM + EVs showed diagnostic differences for AD, and perhaps fronto-temporal dementia, multiple sclerosis, and TBI. Regarding AD, patients have much higher p-T181-tau, p-S396-tau, and Aβ42 (pathogenic proteins for tangle and plaque pathology), as well as Ser-phosphorylated insulin receptor substrate-1 (IRS-1), and lysosomal enzymes [53–55]. Ongoing studies based on samples from large-scale longitudinal studies will determine full diagnostic and predictive potential. According to Dr. Kapogiannis, the key challenges for EV-based diagnostic biomarkers for neurological diseases are (1) elucidating factors (at the levels of EV cargo, endothelial/astrocytic/immune cells) that determine which subset of brain EVs cross the BBB and whether active selection occurs; (2) factors determining rates of transfer and clearance; (3) intra subject variance of EV-derived biomarkers over time; and (4) variability of cargo molecules within an EV population.

#### Human epidermal growth factor receptor 2-targeted extracellular vesicles delivery of therapeutic mRNA for enzyme Prodrug therapy

Human Epidermal Growth Factor Receptor 2 (HER2) is overexpressed in aggressive breast cancers and metastasis to the brain is a major complication of breast cancer. Dr. A.C. Matin and coworkers have constructed human DC-derived exosomes capable of specifically targeting HER2 positive cancer. By using EVs as the “body’s antigen delivery system” for targeting a novel prodrug 6-chloro-9-nitro-5-oxo-5H-benzo(a)phenoxazine (CNOB)/ChrR6 regimen specifically to HER2 positive cancer, the cytotoxic product of this regimen, 9-Amino-6-chloro-5H-benzo(a)phenoxazine-5-one (MCHB), can be visualized noninvasively in living mice (Fig. 3e) [56–59]. These exosomes confer CNOB activating capability specifically on HER2 positive cells [unpublished], and can also deliver genes in vivo [60]. A cranial window model in mice showed that EVs can cross the cancerous BBI. Using a HER2 positive cell line that metastasizes to the brain, Dr. Matin’s group is testing the efficacy of the directed exosomes and CNOB to treat brain tumors. These approaches have the potential to treat any cancer overexpressing a marker and by using patient-specific DCs, EV-based therapy holds the promise of personalized medicine.

### Keynote presentation II: from blood–brain barrier to blood–brain interface: new opportunities for CNS drug delivery

Dr. William Banks emphasized that the BBB acts as a complex interface regulating the exchange of nutrients, informational molecules, and other substances between the CNS and blood. The properties of this interface are not only due to the physical aspects of the barrier, but also to enzymatic activity and brain-to-blood transporters. Dr. Banks noted that the BBB also serves the nutritional, homeostatic, and communication needs of the CNS. For example, BBB transporters for gastrointestinal peptides are key to the gut-brain axis, and BBB transport and secretion of cytokines/chemokines and regulation of CNS immune cell trafficking are key in the neuroimmune axis. The BBB communicates with pericytes, astrocytes, and other cells forming the neurovascular unit. This communication informs the BBB as the needs of the CNS change with, for example, development, fasting, or sleep. Similarly, the BBB adapts to diseases, but maladaptation or malfunction of the BBB is involved in several diseases, including multiple sclerosis, AD, TBI, and obesity. This complexity of the BBB provides a wealth of special opportunities for drug delivery. For example, (1) BBB transport of phosphorothiolate ONT antisenses which can decrease amyloid protein precursor levels, reversing memory impairments in mouse models of AD, (2) pegylated blockers of leptin transport can induce feeding for potential treatment of anorexia, and (3) exosomes can deliver neurotrophins across the BBB for treating neurodegenerative diseases. Dr. Banks concluded that understanding how the BBIs between the CNS and peripheral tissues interact provides unique approaches for CNS drug development [61–65].

### Next generation in vitro BBB models session (Fig. 4)

#### Recapitulating the neurovascular unit in vitro

The BBB, within the neurovascular unit, comprises approximately 600 km of capillaries formed by highly specialized endothelial cells. The neurovascular unit is supported by pericytes that modulate contractility, and astrocytes that regulate capillary dilation. Together these cells regulate the supply of nutrients and other essential molecules to the brain while maintaining tight control of the microenvironment where neurons and other brain cells function.

**Fig. 4:**
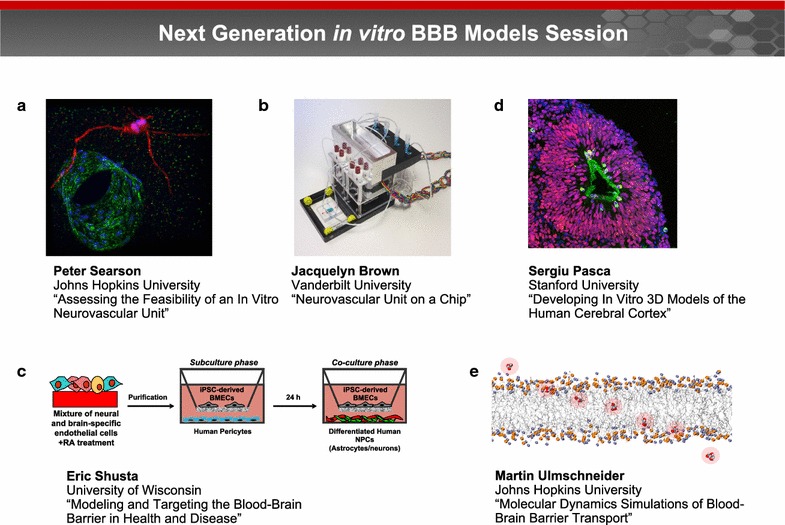
Next generation in vitro BBB models session. Next generation BBB models span organ-on-a-chip devices and models exploiting organogenesis, microfluidics, 3D printing, and self-organization. These models are enabled by advances in human brain-specific cell lines and tissue engineering, particularly in 3D co-culture and matrix materials. **a** Dr. Peter Searson noted that stem cell technology has the potential to overcome the limitations of animal-derived cells and immortalized cell lines, and provide a source of human brain-specific microvascular endothelial cells, astrocytes, and pericytes. induced pluripotent stem cells (iPSCs) can be used to generate human brain microvascular endothelial cells (hBMECs) for developing perfusable brain capillary networks that capture the important physical and biological characteristics of the BBB in a physiologically relevant geometry. Image courtesy of Dr. Searson. **b** Dr. Jacquelyn Brown and colleagues developed a transwell assay that consists of a monolayer of endothelial cells seeded on a porous membrane that is separated into apical and basolateral chambers on either side of the membrane. These platforms have perfusable vascular and brain chambers on either side of the membrane that can be used to measure solute permeability and asses brain penetration. Image courtesy of Dr. Brown. **c** Dr. Eric Shusta reported how endothelial cells generated from human pluripotent stem cells (hPSCs) can be programmed to possess many BBB attributes, including well-organized tight junctions, polarized efflux transport, and nutrient transporter expression. Image courtesy of Dr. Shusta. **d** Dr. Sergiu Pasca discussed how spheroids of pluripotent stem cells (PSCs) can be programmed to form structures that resemble the human cerebral cortex. Image courtesy of Dr. Pasca. **e** Advances in computational methods have enabled a new generation of tools for modeling BBB transport. Dr. Martin Ulmschneider described how these models can simulate spontaneous transmembrane diffusion of small molecules using unbiased long timescale atomic detail molecular dynamics (MD) techniques. Image courtesy of Dr. Ulmschneider

The major barriers to achieving a perfusable in vitro model of the human BBB are a source of relevant cells and 3D cell culture methods to build the model [66]. Dr. Peter Searson noted that stem cell technology has the potential to overcome the limitations of animal-derived cells and immortalized cell lines, and provide a source of human brain-specific microvascular endothelial cells, astrocytes, and pericytes (Fig. 4a). Advances in tissue engineering provide new tools for self-organization of perfusable vascular networks that will provide the foundations for tissue engineered BBB models [67]. For example, the characteristic star-shaped morphology of human astrocytes and the low levels of activation associated with the quiescent state can be recapitulated in novel 3D matrices [68]. Similarly, brain microvascular endothelial cells resist elongation due to shear flow, which may be important in allowing the cells to wrap around and form tight junctions with themselves in brain capillaries [69, 70]. Dr. Searson concluded that these approaches have potential for developing perfusable brain capillary networks that capture the important physical and biological characteristics of the BBB in a physiologically relevant geometry.

#### Neurovascular unit on a chip: new direction in blood–brain barrier modeling and perfusion

Dr. Jacquelyn Brown described how integrating a monolayer of endothelial cells into a microfluidic platform can form the basis for a new generation of brain-on-a-chip devices (Fig. 4b) [71]. Dr. Brown and colleagues developed a transwell assay that consists of a monolayer of endothelial cells seeded on a porous membrane that is separated into apical and basolateral chambers on either side of the membrane. These platforms have perfusable vascular and brain chambers on either side of the membrane that can be used to measure solute permeability and asses brain penetration. Incorporation of a hydrogel layer on the brain-side of the membrane allows co-culture with astrocytes and pericytes and cell–cell communication with the endothelial cells. This dual-chamber device also provides the high shear forces created by flow for mature tight junction formation in the vascular chamber, while keeping shear stress low in the brain chamber. This next generation brain-on-a-chip device has sufficient cell mass to support a breadth of analytical measurements and is a promising tool for BBB modeling and personalized therapy development.

#### Modeling and targeting the blood–brain barrier in health and disease

A renewable cell source for human BBB models has the potential to accelerate brain research and pharmaceutical development. Dr. Eric Shusta reported how endothelial cells generated from human pluripotent stem cells (hPSCs) can be programmed to possess many BBB attributes, including well-organized tight junctions, polarized efflux transport, and nutrient transporter expression (Fig. 4c) [72–76]. Importantly, hPSC-derived BBB endothelial cells respond to cues provided by other cells of the neurovascular unit such as human pericytes, astrocytes, and neurons, to generate very tight barrier properties as measured by transendothelial electrical resistance, while exhibiting molecular permeability that correlates well with in vivo brain uptake. Dr. Shusta suggested that using BBB cells derived from patient induced pluripotent stem cell lines is compatible with disease modeling. These cells can be employed for isogenic modeling of the neurovascular unit and evaluation of experimental drug permeability attributes.

#### Developing tridimensional models of the human cerebral cortex in vitro

Organogenesis involves the self-organization of progenitors and their derivatives into 3D structures of higher cellular complexity. In recent years, 3D culture methods deriving tissue-specific spheroids or organoids from pluripotent stem cells (PSC) have been shown to recapitulate some of these complex cell–cell interactions and tissue cyto-architectures, in contrast to conventional monolayer culture. Dr. Sergiu Pasca discussed how spheroids of PSCs can be programmed to form structures that resemble the human cerebral cortex (Fig. 4d). These floating spheroids grow up to 5 mm in diameter, and include deep and superficial layer pyramidal neurons of the cortex, as well as astrocytes [77, 78]. After developing in culture for ~10 weeks, cortical spheroids display transcriptional characteristics of late mid-fetal human cortex. The emerging neural network activity consists of spontaneous electrical activity, forming functional synapses that can be probed in preparations similar to slice recordings of the animal brain. According to Dr. Pasca, neural spheroid cultures allow detailed examination of human cortical development, function, and disease, and represent a versatile platform for generating other neuronal and glial subtypes in vitro.

#### Molecular dynamics simulations of blood–brain barrier transport

Advances in computational methods have enabled a new generation of tools for modeling BBB transport. Dr. Martin Ulmschneider described how these models can simulate spontaneous transmembrane diffusion of small molecules using unbiased long timescale atomic detail molecular dynamics (MD) techniques (Fig. 4e) [79–81]. According to Ulmschneider, the key advantage of this approach is that it not only provides the free energy barrier profile for molecular diffusion across both membrane types, but also reveals the molecular transport mechanisms, and allows direct determination of the transport kinetics.

### Blood–brain barrier delivery and targeting session (Fig. 5)

#### Overcoming blood–brain barrier for precise diagnosis, targeting and treatment of primary and metastatic brain tumors

**Fig. 5:**
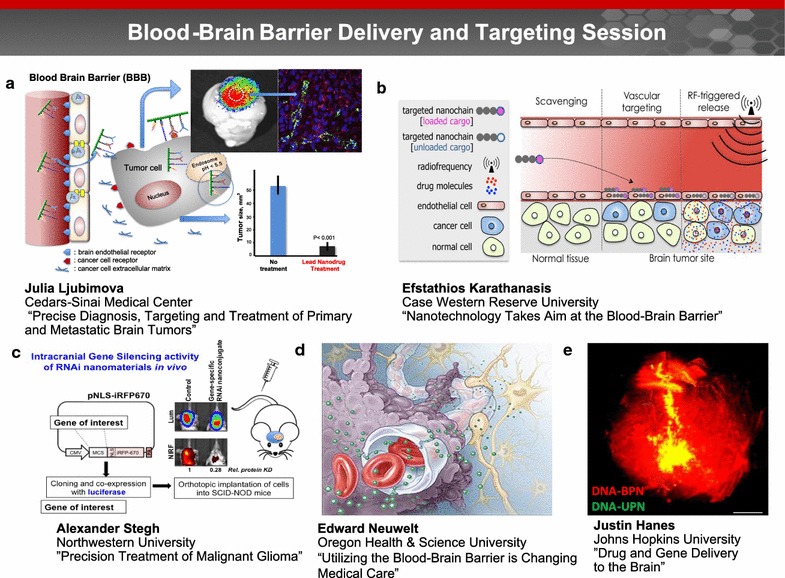
Blood brain barrier delivery and targeting session. Drug delivery through the BBB to the brain can be enhanced utilizing nanoparticles that are designed to exhibit BBB targeting and/or penetrating capabilities. Evidence of these nanoparticles across the BBB can be accessed via in vivo imaging. **a** Dr. Julia Ljubimova presented a novel nanotechnology which can overcome the BBB for precise diagnosis, targeting and treatment of primary and metastatic brain tumors. Use of systemically administered novel nanobiopolymers based on a combination of a polymalic acid platform (Polycefin™ family of nano agents), nano drugs and imaging agents, dramatically reduced tumor size by 90% and normalized brain cancer vasculature. Image courtesy of Dr. Ljubimova. **b** Dr. Karathanasis’ group has developed a new class of multicomponent chain-like nanoparticles, termed nanochains. Due to enhanced site-specific targeting and radiofrequency-triggered drug release, the nanochains facilitate effective delivery of drugs across the BBB into hard-to-reach brain tumors (image reprinted with permission from [91]). **c** Dr. Alexander Stegh and his team developed RNAi-based Spherical Nucleic Acids (SNAs) for the treatment of Glioblastoma multiforme (GBM). Upon IV administration, SNAs cross the BBB and disseminate within intracranial patient-derived xenografts and genetically engineered mouse model tumors. Image courtesy of Dr. Stegh. **d** Dr. Edward Neuwelt emphasized that advances toward penetrating the BBB must consider both normal and abnormal function, as well as the entire neurovascular unit (illustrated here). Image courtesy of Dr. Neuwelt. **e** Dr. Justin Hanes presented a nanoparticle-based platform for drug delivery to the brain. Brain-penetrating DNA nanoparticles (*red color*) spread throughout the entire rat striatum, as compared to standard DNA nanoparticles (*yellow color*) that do not spread as well. Image courtesy of Dr. Panagiotis Mastorakos and the work is a collaboration between Dr. Hanes’ research group and that of Prof. Jung Soo Suk

Dr. Julia Ljubimova presented a novel nanotechnology which can overcome the BBB for precise diagnosis, targeting and treatment of primary and metastatic brain tumors. Use of systemically administered novel nanobiopolymers based on a combination of a polymalic acid platform (Polycefin™ family of nano agents), nano drugs and imaging agents, dramatically reduced tumor size by 90% and normalized brain cancer vasculature (Fig. 5a). Such nano drug treatments also significantly protected the brain from edema development [82]. Polycefin nano drug variants were also engineered to treat human Epidermal Growth Factor Receptor (EGFR)-positive lung cancer, triple negative breast cancer, and HER-2/*neu* positive breast tumors in nude mice. The same nano drugs were used to treat brain metastases from lung, triple negative and HER2/*neu* positive breast cancer in a mouse model [83–85]. Further, HER2/*neu* positive breast cancer was treated with a combination nanodrug that blocked HER2/*neu* synthesis and provided an immune system boost by tumor-targeted IL-2 [86]. According to Dr. Ljubimova, these data suggest that nanotechnology can be harnessed for multiple therapeutic interventions.

#### Nanotechnology takes aim at the blood–brain barrier

In his discussion, Dr. Efstathios Karathanasis noted that aggressive efforts are currently underway to further understand brain tumor’s microenvironment and identify brain tumor cell-specific regulators amenable to pharmacologic interventions. While potent, new agents are continuously becoming available, efficient drug delivery to brain tumors remains a limiting factor. This stems from the fact that drug molecules are not specifically designed to overcome the microenvironment of hard-to-treat cancers. To effectively seek and destroy brain tumors, Dr. Karathanasis’ group has developed a new class of multicomponent chain-like nanoparticles, termed nanochains [87]. A nanochain particle is comprised of three iron oxide nanospheres and a drug-loaded liposome chemically linked into a 100-nm linear, chain-like assembly. Due to enhanced site-specific targeting and radiofrequency-triggered drug release [88–90], the nanochains facilitate effective delivery of drugs across the BBB into hard-to-reach brain tumors [87], allowing significant expansion of therapies to diseases in the brain where success is currently limited (Fig. 5b) [91].

#### Spherical nucleic acids for the precision treatment of malignant glioma

Glioblastoma multiforme (GBM) is an incurable cancer, with one of the poorest survival rates of just 14–16 months after diagnosis [92]. Dr. Alexandar Stegh noted that an incomplete understanding of how genetic aberrations influence GBM tumor response to therapy, combined with the lack of effective delivery of drugs to the CNS, have contributed to making GBM one of the most difficult cancers to treat. Dr. Stegh and his team developed RNAi-based Spherical Nucleic Acids (SNAs) for the treatment of GBM. Upon IV administration, SNAs cross the BBB and disseminate within intracranial patient-derived xenografts and genetically engineered mouse model tumors. SNAs potently regulate gene expression via RNA interference, reduce GBM burden, and increase animal survival (Fig. 5c) [93–95]. Dr. Stegh’s group has conducted toxicology studies in non-human primates, which will lead to an Investigational New Drug (IND) application and a phase 0 clinical trial in the spring of 2017.

#### Three areas where studies of the blood–brain barrier change patient care

Current BBB research is focused on improving long term outcomes in patients with primary and metastatic brain tumors. Dr. Edward Neuwelt presented three major research areas: (1) Intra-arterial hyperosmotic mannitol infusion utilizing BBBD transiently increases delivery of chemotherapy, antibodies, and nanoparticles to the brain. Multiple clinical trials have shown its safety and efficacy in methotrexate-based chemotherapy in conjunction with BBBD, particularly in patients with newly diagnosed primary CNS lymphoma [96]. Prospective evaluation of survivors who were treated with BBBD showed stable or improved cognitive status at a median follow-up of 12 years [97]; (2) Neuroimaging with the iron nanoparticle ferumoxytol provides a mechanism to differentiate tumor progression from pseudoprogression, which can resolve the clinical dilemma of continuation of effective therapy or transferring non-responders to novel therapies [98]; and (3) In conjunction with BBBD, thiols, NAC and sodium thiosulfate can protect against cisplatin-induced hearing loss and severe thrombocytopenia [99, 100]. Dr. Neuwelt concluded that advances toward penetrating the BBB must consider both normal and abnormal function, as well as the entire neurovascular unit (Fig. 5d).

#### Drug and nucleic acid delivery to the brain

Dr. Justin Hanes presented a nanoparticle-based platform for drug delivery to the brain. By analyzing the movements of nanoparticles of various diameters and surface coatings within fresh human and rat brain tissue, his lab discovered that sub-114 nm nanoparticles with a dense surface coverage of polyethylene glycol (PEG) can rapidly penetrate both healthy and tumor brain tissue [101]. Such brain penetrating nanoparticles (BPN) can be used to carry drug or nucleic acid to treat brain tumors following local injection [102]. In addition to local injection, DNA carrying BPN was shown to effectively distribute within the brain following convection enhanced delivery and result in overall level of transgene expression (Fig. 5e). This work was a collaboration between Dr. Hanes’ research group and that of Prof. Jung Soo Suk [103–105]. More recently, Dr. Hanes’ group collaborated with Dr. Richard Price and colleagues at the University of Virginia to show that systemically-administered BPN can be delivered into desired regions of the brain using image-guided focused ultrasound with microbubbles that temporarily disrupt the BBB [106].

## Workshop conclusions

In both health and disease, endothelial cells, neurons, pericytes, and glia constitute a neurovascular unit that regulates the BBB. Bi-directional accessibility and transport of molecules between the blood and brain are dynamically regulated in response to a number of events, such as exercise, stress, and sleep. The integrity of BBB deteriorates with age, and BBB breakdown can lead to entry of neurotoxic blood-derived products to the brain causing inflammation, neuronal injury and neurodegeneration. Sleep-wake disturbances and circadian dysregulation can further affect the neurovascular unit and BBB integrity. Genetic risk factors for AD together with vascular and environmental factors act on blood vessels in the brain resulting in BBB breakdown and dysfunction (e.g., faulty clearance of Amyloid beta). Changes in CBF following TBI, in addition to vascular pathology, can result in BBB dysfunction.

The BBB also poses a tremendous hurdle for systemic delivery of drugs to the brain. Multifunctional imaging and treatment agents utilizing nanoparticles, including EVs, are being designed and tested to cross the BBB through receptor mediated transcytosis and to deliver drugs to brain cells efficiently and safely. Among various delivery routes, intranasal delivery appears to be a promising route to deliver drugs into the brain. Future scientific and clinical developments in treating primary and secondary brain tumors are needed to improve our understanding of targeted brain delivery through the blood and blood-based biomarker development. In vitro models of the neurovascular unit allow us to elucidate the underlying physical and biochemical processes that regulate the BBB, including brain capillaries and microvessels, while simultaneously allowing us to systematically increase the complexity of the model to incorporate the blood component and generate a neuro-vascular-blood unit. Advances in BBB modeling, targeting and therapeutic delivery must also account for both normal and abnormal functions of the neuro-vascular-blood unit.

## Research needs and challenges

To date, the role of blood in the BBI (e.g., blood-derived factors, blood-based biomarkers, circulating exosomes) in the pathogenesis of neurological disorders and brain injury states (e.g., brain trauma, stroke, amyotrophic lateral sclerosis, multiple sclerosis, AD) and the underlying neurovascular mechanisms remain largely unknown and under-researched. Speaker presentations and panel discussions highlighted the following overarching research needs and challenges across the interface between the blood and the brain. These can be grouped into three categories:

1. Biophysical/biochemical properties of the BBB
2. Standardize procedures to assess changes in the BBB, employing methods that more accurately reflect changes in physiological analytes and relevant clinical conditions.
3. Develop benchmarks and validate the best practices for models of the neurovascular unit and to incorporate the blood component as an integral part of the neurovascular unit.
4. Determine reproducible protocols for differentiation of iPS cells into brain-specific endothelial cells, astrocytes, and pericytes for cross-laboratory investigations of the neurovascular unit, including the blood component.
5. Develop a consensus “gold standard” approach for BBB-opening for delivery of therapeutics from the blood to the brain.
6. Utilization of EVs in diagnostics and treatments
7. Develop standardized methods to track how exosomes and/or their cargo enter and leave the brain.
8. Develop protocols to stimulate parent cells to produce exosomes of interest with GMP standards.
9. Develop strategies to efficiently load therapeutic cargo into exosomes.
10. Develop methodologies to reduce immunogenicity of exosomes.
11. Optimize methods to deliver exosomes to the brain and target specific cells types.
12. Biomarker discoveries
13. Develop a comprehensive proteomics and RNA sequencing (RNA-Seq) molecular atlas across the BBB in animals and humans for new targets, signaling pathways within the neurovascular unit, drug screens, generation of new transgenic models and new therapies.
14. Determine the origin of extracellular RNA (exRNA) and other biomolecules in peripheral biofluid samples.

### Recommendations and opportunities

The following recommendations and opportunities were noted by the speakers:

- Membrane transporters can be capitalized on to optimize brain exposure of potentially neuroprotective compounds in TBI and ischemic brain injury; repurposing transporter inhibitors/inducers should expedite clinical translation of such treatment strategies.
- MPs populations found in peripheral biofluid samples can be a target for assessment of post-TBI or disease progression as well as prophylaxis or amelioration of injury progression.
- Plasma EVs of neural origin can be the source of biomarkers and may provide a unique window for studying organ-specific cellular processes in living humans and to follow responses to experimental treatments. For example, a profile of exosomal peptides may serve as a blood test for AD and other neurological diseases.
- Nanoparticle and EV-based therapies hold the promise of personalized medicine.
- There are opportunities and challenges in developing the building blocks for in vitro models of the neuro-vascular-blood unit. For example, iPSC-derived brain microvascular endothelial cells can be used as a research tool to explore drug permeability and disease at the human BBB.
- Isogenic patient-derived models of the neurovascular unit can be used for biomarker identification, the study of disease progression, and therapeutic testing.
- Patient-specific neurovascular unit models may complement or replace animal models.
- In silico predictive modeling is advancing rapidly and presenting new exciting opportunities to capture many biological processes.

As our understanding of the complex interactions among the various cellular and molecular components of the BBB and the interdependency of the brain and other organ systems with BBB function grows, more opportunities will reveal themselves to stimulate basic and translational research for developing new diagnostic and treatment modalities related to the neuro-vascular-blood unit.

## Additional file

**Additional file 1.** 2016 Blood Brain Interface Workshop Agenda.

## Abbreviations

AD: Alzheimer’s disease
BBB: blood–brain barrier
BBBD: blood–brain barrier disruption
BBI: blood–brain interface
BPN: brain penetrating nanoparticles
CBF: cerebral blood flow
CNS: central nervous system
CSF: cerebrospinal fluid
DC: dendritic cell
EV: extracellular vesicle
exRNA: extracellular RNA
GBM: glioblastoma multiforme
GNV: grapefruit derived nanovector
MP: microparticle
MRgFUS: magnetic resonance-guided focused ultrasound
NAC: *N*-acetyl cysteine
ONT: oligonucleotide
PEG: polyethylene glycol
PSC: pluripotent stem cells
SDC: interferon gamma-stimulated dendritic cell
SDC-exos: interferon gamma-stimulated dendritic cell-derived exosomes
SNAs: spherical nucleic acids
TBI: traumatic brain injury
