# Supplementary material for: NIH workshop report on the trans-agency blood–brain interface workshop 2016: exploring key challenges and opportunities associated with the blood, brain and their interface
Source: Fluids Barriers CNS. 2017 May 1;14:12. doi: 10.1186/s12987-017-0061-6 (PMC5410699; doi:10.1186/s12987-017-0061-6)
Supplement: Supplementary file 1 — Additional file 1. 2016 Blood Brain Interface Workshop Agenda. [file 12987_2017_61_MOESM1_ESM.pdf]

AGENDA, Tuesday, June 7

7:30 AM ***Welcome and Registration***

General Session (Conference Room C/D, 1st Floor)

8:00 AM ***Welcome***
Gary Gibbons, MD, Director, NHLBI

8:10 AM ***Workshop Introduction***
Keith Hoots, MD, Director, DBDR

Goals and Objectives
Margaret Ochocinska, PhD, Program Director, DBDR

8:20 AM ***Keynote: Initial Experience in a Pilot Study of Blood-Brain Barrier Opening for Chemo-Drug Delivery to Brain Tumors by MR-Guided Focused Ultrasound***
Todd Mainprize, MD, Sunnybrook Health Sciences Center

Blood Sciences Session – Part I (Conference Room C/D, 1st Floor)
Session Chair: Berislav Zlokovic, MD, PhD, University of Southern California

8:40 AM ***Session Introduction***
Berislav Zlokovic, MD, PhD, University of Southern California

8:45 AM ***The Blood-Brain Barrier and Neurodegeneration***
Berislav Zlokovic, MD, PhD, University of Southern California

9:05 AM ***Microfluidics for Blood Research: from disease simulation to patient-specific phenotyping to diagnostics***
Scott Diamond, PhD, Penn Center for Molecular Discovery

9:25 AM ***Microparticles Impact Coagulation after Traumatic Brain Injury***
Michael Goodman, MD, University of Cincinnati

9:45 AM ***Fibrinogen in Neurological Diseases: mechanisms, imaging, therapeutics***
Katerina Akassoglou, PhD, UCSF School of Medicine

10:05 AM ***Exosomes in Glioma: their potential as carriers of information between the tumor and immune cells***
Theresa Whiteside, PhD, University of Pittsburgh

10:25 AM ***Break***

Blood Sciences Session – Part II (Conference Room C/D, 1st Floor)
Session Chair: A Tamara Crowder, PhD, Combat Casualty Care Research Program, DoD

10:40 AM ***Session Introduction***
A Tamara Crowder, PhD, Combat Casualty Care Research Program, DoD

10:50 AM ***Monitoring the Central Nervous System through Peripheral Biofluids***
Kendall Jensen, PhD, TGen Center for Noninvasive Diagnostics

11:10 AM ***Studying the Blood-Brain Barrier: perspectives from understanding the biokinetics of biomarkers of brain injury***
Alex Valadka, PhD, Virginia Commonwealth University

11:30 AM ***Post-traumatic Cerebral Blood Flow, Autoregulation, and the Neurovascular Unit***
Donald Marion, MD, Defense and Veterans Brain Injury Center

11:50 AM ***Employing Transporters at Blood-Brain Interfaces to Regulate the Brain's Metabolomic and Pharmacologic Microenvironment***
Robert Clark, MD, University of Pittsburgh

12:10 PM ***Lunch***

Exosome Therapeutics Session (Conference Room C/D, 1st Floor)
Session Chair: Richard Kraig, MD, PhD, University of Chicago Medical Center

1:10 PM ***Session Introduction***
Richard Kraig, MD, PhD, University of Chicago Medical Center

1:15 PM ***In Vivo Tracking of Dendritic Cell Exosomes Delivered to Brain***
Richard Kraig, MD, PhD, University of Chicago Medical Center

1:35 PM ***High Content Proteomics/Lipidomics Analysis: on a path toward understanding the mechanisms of exosome-mediated cellular uptake and blood-brain barrier crossing***
Anastasia Khvorova, PhD, University of Massachusetts Medical School

1:55 PM ***Exosome-like Nanoparticles Delivering Therapeutic Agents through an Intranasal Route Inhibit Brain Tumor Progression***
Huang-Ge Zhang, PhD, University of Louisville

2:15 PM ***Plasma Exosomes Enriched for Neuronal Origin: a source of biomarkers for neurodegenerative and neuroinflammatory diseases***
Dimitrios Kapogiannis, MD, National Institute of Aging, NIH

2:35 PM ***HER2-targeted Extracellular Vesicles Delivery of Therapeutic mRNA for Enzyme Prodrug Therapy***
A.C. Matin, PhD, Stanford University

2:55 PM ***Break***

Discussion Session

3:10 PM ***Open Microphone Discussion and Panel - Blood Brain Interface I***
Moderator: Andrei Kindzelski, MD, PhD, Program Director, DBDR

4:45 PM ***Wrap-up***
Margaret Ochocinska, PhD

5:00 PM **Adjourn**

6:30 PM **Informal Dinner**
Democracy Grille

Wednesday, June 8

7:30 AM ***Welcome and Registration***

General Session (Conference Room C/D, 1st Floor)

8:00 AM ***Keynote: From Blood–Brain Barrier to Blood–Brain Interface: new opportunities for CNS drug delivery***
William Banks, MD, FACE, University of Washington

Next Generation *in vitro* BBB Models Session (Conference Room C/D, 1st Floor)
Session Chair: Peter Searson, PhD, Johns Hopkins School of Medicine

8:20 AM ***Session Introduction***
Peter Searson, PhD, Johns Hopkins School of Medicine

8:25 AM ***Assessing the Feasibility of an in vitro Neurovascular Unit***
Peter Searson, PhD, Johns Hopkins School of Medicine

8:45 AM ***NeuroVascular Unit (NVU) on a Chip: new direction in blood-brain barrier modeling and perfusion***
Jacquelyn Brown, PhD, Vanderbilt University

9:05 AM ***Modeling and Targeting the Blood-Brain Barrier in Health and Disease***
Eric Shusta, PhD, University of Wisconsin - Madison

9:25 AM ***Developing Tridimensional Models of the Human Cerebral Cortex in vitro***
Sergiu Pasca, MD, Stanford University

9:45 AM ***Revealing the Transport Mechanisms, Kinetics, and Energetics of Drugs Diffusing through Membranes of the Blood-Brain Barrier***
Martin Ulmschneider, PhD, Johns Hopkins University

10:05 AM ***Break***

Blood-Brain Barrier Delivery and Targeting Session (Conference Room C/D, 1st Floor)
Session Chair: Julia Ljubimova, MD, PhD, Cedars-Sinai Medical Center

10:20 AM ***Session Introduction***
Julia Ljubimova, MD, PhD, Cedars-Sinai Medical Center

10:25 AM ***Overcoming Blood-Brain Barrier for Precise Diagnosis, Targeting and Treatment of Primary and Metastatic Brain Tumors***
Julia Ljubimova, MD, PhD, Cedars-Sinai Medical Center

10:45 AM ***Nanotechnology Takes Aim at the Blood-Brain Barrier***
Efstathios (Stathis) Karathanasis, PhD, Case Western Reserve University

11:05 AM ***Spherical Nucleic Acids for the Precision Treatment of Malignant Glioma***
Alexander Stegh, PhD, Northwestern University

11:25 AM ***Three Areas Where Studies of the Blood-Brain Barrier Change Patient Care***
Edward Neuwelt, MD, Oregon Health & Science University and the Portland Veterans Affairs Medical Center

11:45 AM ***Drug and Nucleic Acid Delivery to the Brain***
Justin Hanes, PhD, Johns Hopkins University

12:05 AM ***Break***

Discussion Session

12:15 PM ***Open Microphone Discussion and Panel - Blood Brain Interface II* (Conference Room C/D, 1st Floor)**
Moderator: Christina Liu, PhD, Program Director, NCI

1:15 PM ***Wrap Up and Next Steps***
Margaret Ochocinska, PhD

1:30 PM **Adjourn Workshop**
